# Middle Ear Barotrauma among Licensed Para-pilots of a Metropolitan City: A Descriptive Cross-sectional Study

**DOI:** 10.31729/jnma.7257

**Published:** 2022-06-30

**Authors:** Biraj Baral, Deepak Regmi, Aakriti Dawadi, Bibek Adhikari

**Affiliations:** 1 Kathmandu Medical College and Teaching Hospital, Sinnamangal, Kathmandu, Nepal; 2 Department of ENT, Kathmandu Medical College and Teaching Hospital, Sinnamangal, Kathmandu, Nepal; 3 Maharajgunj Medical Campus, Maharajgunj, Kathmandu, Nepal; 4 Islington College, Kamalpokhari, Kathmandu, Nepal

**Keywords:** *barotrauma*, *eustachian tube*, *Nepal*, *pilots*

## Abstract

**Introduction:**

Middle ear barotrauma is a tissue injury to the ear secondary to inadequate pressure equalisation between the middle ear and the external environment. Paragliding, though an exciting sport, has its own risks and hazards. Para-pilots experience a variety of ear-related symptoms due to pressure discrepancies between the middle ear and ambient air. Middle ear barotrauma amongst para-pilots is a common yet neglected problem. The aim of this study was to find the prevalence of middle ear barotrauma among licensed para-pilots of a metropolitan city.

**Methods:**

A descriptive cross-sectional study was conducted amongst para-pilots practising in different paragliding companies in a metropolitan city. The study was conducted from 10^th^ October, 2021 to 22^nd^ October, 2021 after getting ethical approval from the Institutional Review Committee (Reference number: 0410202109/2021). A sample size of 76 participants was taken using convenience sampling technique. Data was collected from participants after performing an otoscope examination. The data were entered into Microsoft Excel version 2016 and analysed using the Statistical Package for the Social Science Version 22.0. Point estimate at a 95% Confidence Interval was calculated along with frequency and proportion for binary data and mean and standard deviation for continuous data.

**Results:**

Out of 76 participants, the prevalence of middle ear barotrauma was 10 (13.2%) (5.58-20.81 at 95% Confidence Interval).

**Conclusions:**

The prevalence of middle ear barotrauma was similar to other studies done in similar settings.

## INTRODUCTION

The inflammatory change of the tympanic membrane and middle ear cavity from pressure discrepancies between the middle ear cavity and the atmospheric air, which may occur during aviation, diving, or hyperbaric oxygen therapy, is referred to as middle ear barotrauma.^1^ Sports with extreme changes in atmospheric pressure such as paragliding commonly place the athlete at risk for barotrauma injuries, especially in the middle ear.^2,3^

Para-pilots practice cross country flights and Pokhara discovery flights where they are exposed daily to the rapid change in altitude and air pressure.^3^ Despite all the risks it carries, no reports are present in the current context reflecting them. Therefore extensive research and knowledge are required in this field. This study uses Teed's classification as a method to diagnose and grade barotrauma occurrence as suggested by prior research conducted on middle ear barotrauma.^4,5^

The aim of this study was to find the prevalence of middle ear barotrauma among licensed para-pilots of a metropolitan city.

## METHODS

This descriptive cross-sectional study was conducted from 10^th^ October, 2021 to 22^nd^ October, 2021 in Pokhara, Nepal. Ethical approval was taken from the Institutional Review Committee of Kathmandu Medical College (Reference number: 0410202109/2021). Only licensed para-pilots were included in the study. The samples having impacted ceruminous wax in external auditory meatus causing poor visibility of the tympanic membrane were excluded. Convenience sampling was done. The sample size was calculated using the formula:


$$\begin{array}{ll} \text{n} & ={\text{Z}}^{2}\times \frac{\text{p}\times \text{q}}{{\text{e}}^{\text{2}}}\\ & =1.96^{2}\times \frac{0.5\times 0.5}{0.1^{2}}\\ & =96 \end{array}$$

Where,

n = minimum required sample size
Z = 1.96 at 95% Confidence Interval (CI)
p = prevalence taken as 50% for maximum sample size calculation
q = 1-p
e = margin of error, 10%

As the total number of registered licensed para-pilots in Pokhara was 356 at the time of study,^6^ the above sample size was adjusted for finite population as:


$$\begin{array}{ll} \text{n}’ & =\frac{\text{n}}{\left[1+\frac{\text{n}-1}{\text{N}}\right]}\\ & =\frac{\text{96}}{\left[1+\frac{\text{96}-1}{\text{356}}\right]}\\ & =76 \end{array}$$

Where,

n' = adjusted sample size for finite population
N = total number of para-pilots in Pokhara, 356^6^

The final sample size was 76. Data were collected by performing an otoscopy examination after taking informed written consent, for which Teed Grading was used as suggested by prior studies.^4,5^ Studies on middle ear barotrauma show that Teed Grading of 0 was not included in the prevalence of middle ear barotrauma while the Teed Grading of 1 to 4 were included in the prevalence study.^4,5^

The collected data were entered using Microsoft Excel version 2016 and statistically analysed using the Statistical Package for the Social Sciences version 22.0. Point estimate at 95% Confidence Interval (CI) was calculated along with frequency and proportion for binary data and mean with standard deviation for continuous data.

## RESULTS

Among the 76 participants, the prevalence of middle ear barotrauma was 10 (13.15%) (5.58-20.81 at 95% Confidence Interval). The exceptions were unilateral cases which were not met by three bilateral cases with Teed grading of 1,14,4, and 4,1 on right and left ears, respectively. For these cases, maximum grading of Teed was taken. On otoscopic examination, 3 (30.00%) respondents had grade 4, 1 (1.00%) had grade 2, and 6 (6.00%) had grade 1 tauma. Grade 3 Teed were not evident in any of the cases. All 10 (13.15%) barotraumatic cases were found in males.

Equal cases of middle ear barotrauma, five (50.00%), were reported from age groups 21-30 and 31-40 years. None of the participants fell under the age groups of less than 20 or more than 41 years (Table 1).

**Table 1:** Distribution of participants by age (n = 10).

Characteristics		Teed grade			Total n (%)
		1 n (%)	2 n (%)	4 n (%)	
Age (in years)	<20	-	-	-	-
	21-30	4 (66.67)	-	1 (33.33)	5 (50.00)
	31-40	2 (33.33)	1 (100.00)	2 (66.67)	5 (50.00)
	41 +	-	-	-	-

The mean age of para-pilots reported with middle ear barotrauma i.e. Teed >0, was found to be 32±5.34 years. The mean years of experience as a professional licensed para-pilots among the cases was 4.9±2.80 years. Five (50.00%) cases had professional experience of about 4 to 8 years (Table 2).

**Table 2:** Distribution of the grading as per professional experience (n = 10).

Years of registration as a licensed tandem parapilot	Teed grade			Total n (%)
	1 n (%)	2 n (%)	4 n (%)	
1 year to 4 years	2 (33.33)	-	1 (33.33)	3 (30.00)
4 years to 8 years	3 (50.00)	1 (100.00)	1 (33.33)	5 (50.00)
8 years and above	1 (16.67)	-	1 (33.33)	2 (20.00)

The middle ear barotrauma was seen in 9 (90.00%) para-pilots performing cross country flights for less than five times per month. Grade 1 Teed were seen in 6 (60.00%) cases amongst them. For Pokhara discovery flights, a maximum of 5 (50.00%) performed 21-25 flights per month, whereas 4 (40.00%) had Teed grade 1 barotrauma. Two cases (20.00%) had grade 4 Teed for the regularity of 26-30 flights per month (Table 3).

**Table 3:** Distribution of type of flights performed per month with middle ear barotrauma grading (n = 10).

Type of flights performed per month		Teed grade			Total n (%)
		1 n (%)	2 n (%)	4 n (%)	
Cross country flights	≤5	6 (100.00)	1 (100.00)	2 (66.67)	9 (90.00)
	≥6	-	-	1 (33.33)	1 (10.00)
Total		6 (100.00)	1 (100.00)	3 (100.00)	10 (100)
	21-25	4 (66.67)	-	1 (33.33)	5 (50.00)
Pokhara discovery flights	26-30	1 (16.67)	1 (100.00)	2 (66.67)	4 (40.00)
	31-35	1 (16.67)	-	-	1 (10.00)
Total		6 (100.00)	1 (100.00)	3 (100.00)	10 (100.00)

## DISCUSSION

The prevalence of middle ear barotrauma was found to be 13.20% in the present context. In this study, a total of 76 participants were undertaken. People of different ages were practising this adventurous sport as a part of their profession. On otoscopic examination, maximum participants were found to have Teed's grade of 0. For middle ear barotraumatic cases, maximum cases of grade 1 Teed were seen.

The study was conducted to find out the prevalence of middle ear barotrauma amongst para-pilots of Pokhara, Nepal. As a tourism adventure, paragliding is becoming a popular recreational sport. Due to the lack of prior studies on the middle ear barotrauma in the similar settings, this study was conducted in the nearest location where commercial and military para-flights were performed.^7-9^

The prevalence of the middle ear barotrauma in a prior study was obtained as 85%, which is significantly higher than the findings of this study on para-pilots.^7^ The literature review of middle ear barotrauma done in military aircrews showed the prevalence of 12% which is slightly lower than our study on para-pilots.^8^ A study done in aircraft passengers revealed the prevalence of 14% which is slightly higher than our survey.^9^

Paragliding companies claim a change in altitude of 2500 m to 800 m within approximately 20 minutes and also have cross country flights with a change in altitude from 4500 m to 800 m.^10^ Such steep changes in altitude might have created the required change in atmospheric pressure sufficient enough to cause barotrauma symptoms. This assumption is also supported by a similar article.^11^

There are some limitations of the present study. Participants having clean external auditory canal where the tympanic membrane can be easily visualised were taken for the study. The interpretation would be more scientific if all the participants could be examined. The study of prevalence of middle ear barotrauma was only conducted in one municipality and excludes the para-pilots in other parts of the country. Furthermore, due to the descriptive study design, association between variables could not be made.

## CONCLUSIONS

The prevalence of middle ear barotrauma was similar to other studies done in similar settings. In our study, the para-pilots were found to have barotrauma injuries, particularly in the middle ear. In light of this, it is imperative to address this problem, and a need for further study on middle ear barotrauma is observed throughout the study.
